# Transcriptional Regulations on the Low-Temperature-Induced Floral Transition in an *Orchidaceae* Species, *Dendrobium nobile*: An Expressed Sequence Tags Analysis

**DOI:** 10.1155/2012/757801

**Published:** 2012-04-09

**Authors:** Shan Liang, Qing-Sheng Ye, Rui-Hong Li, Jia-Yi Leng, Mei-Ru Li, Xiao-Jing Wang, Hong-Qing Li

**Affiliations:** ^1^Guangdong Provincial Key Laboratory of Biotechnology for Plant Development, School of Life Science, South China Normal University, Guangzhou 510631, China; ^2^Key Laboratory of Plant Resources Conservation and Sustainable Utilization, South China Botanical Garden, Chinese Academy of Sciences, Guangzhou 510650, China

## Abstract

Vernalization-induced flowering is a cold-relevant adaptation in many species, but little is known about the genetic basis behind in *Orchidaceae* species. Here, we reported a collection of 15017 expressed sequence tags (ESTs) from the vernalized axillary buds of an *Orchidaceae* species, *Dendrobium nobile*, which were assembled for 9616 unique gene clusters. Functional enrichment analysis showed that genes in relation to the responses to stresses, especially in the form of low temperatures, and those involving in protein biosynthesis and chromatin assembly were significantly overrepresented during 40 days of vernalization. Additionally, a total of 59 putative flowering-relevant genes were recognized, including those homologous to known key players in vernalization pathways in temperate cereals or *Arabidopsis*, such as cereal *VRN1*, *FT/VRN3*, and *Arabidopsis AGL19*. Results from this study suggest that the networks regulating vernalization-induced floral transition are conserved, but just in a part, in *D. nobile*, temperate cereals, and *Arabidopsis*.

## 1. Introduction

Transition from the vegetative phase to the flowering phase is crucial to both development and reproduction in plants. Besides endogenous signals, this process is also affected by external cues such as day length and temperature. Vernalization, an exposure to low temperature extending over a period differing from species to species, is an adaptive nature to ensure some plants survival in harsh winters and flowers under a favourable condition in spring. In the dicot model *Arabidopsis*, vernalization-regulated flowering is mediated by both *FLC*-dependant and -independent pathways [1, 2], in which the gene *VIN3* (which interacts with *VRN2*), a key upstream component, is probably activated by exposure to low temperature and subsequently leads to changes in histone methylation of downstream gene regions [3]. For example, the expression of *FLC* is suppressed by vernalization through enrichment of H3K27m3 on the chromatin [3], which consequently releases *FT* and *SOC1* from inhibition by FLC to promote the transition to flowering. This *FLC*-dependant pathway is regulated by both temperature and day length [4]. *AGL19*, a close relative of *SOC1*, is believed to mediate an *FLC*-independent pathway that activates flowering under vernalization in *Arabidopsis* [1]. This process is associated with a cold-induced decrease of H3K27m3 on *AGL19 *locus and probably also with the loss of function of CLF and MSI1 or the involved complex [5]. Ectopically expression of *Arabidopsis AGL19* leads to only mild abnormalities, suggesting that *AGL19 *has a limited role in flowering control in *Arabidopsis* [1]. In addition to *FLC* and *AGL19*, *MAF2* and *AGL24* are also involved in the floral transition in *Arabidopsis* [6, 7]. At present, *FLC* is thought to be central to the control of flowering in *Arabidopsis* and probably also in most other dicots [1, 2].

Monocots, however, are likely to be different: no monocot orthologs of *Arabidopsis FLC* have been found so far. In temperate cereals such as wheat and barley, responses to vernalization are mediated by *VRN1*, *VRN2*, and *VRN3* [2]. Cereal *VRN1* has two roles, namely, (a) to transmit the cold signal and thereby induce *VRN3*, the ortholog of *AtFT* in cereals [11] and (b) to act as a floral meristem identify which is activated by *FT/VRN3 *[12]. Thus, *VRN1* and *FT/VRN3* form a positive feedback loop and regulate each other under vernalization. *VRN1* may also have a role in storing the memory of vernalization through methylation of Histone 3 [13], which resembles the epigenetic property of *Arabidopsis FLC*. However, the exact mechanism of this process remains unclear so far. Cereal *VRN2* acts as a flowering repressor that is regulated by both low temperatures and day length [14]. Cold can lower the expression of *VRN2* [15], thereby releasing *FT/VRN3* from repression [16]. *VRN2* may be inhibited by *VRN1* under vernalization [9, 10, 14]. To summarize, vernalization-induced regulation of flowering in monocots is evolutionarily divergent from that in dicots, although the networks are similar to some extent [2].

*Orchidaceae *is the largest family in the plant kingdom and is considered particularly speciational, indicating that *Orchidaceae* species are well adapted to the surrounding environments. *Dendrobium* is a vast genus consisting of more than a thousand species that are native to South Asia, Australia, New Zealand, and Oceania [17]. Species of this genus differ in the extent of dependence on vernalization. For instance, *Dendrobium phalaenopsis* can flower at high temperatures whereas *Dendrobium nobile* requires vernalization and flowers only after a spell of exposure to relatively low temperatures [18]. *D. nobile* is used as a herbal medicine and is popular as a potted ornament prized by growers and hobbyists for its beautiful flowers. Its juvenile period lasts for at least 3–5 years, which makes it difficult to meet the commercial demands. To accelerate flowering, potted *D. nobile* plants are always kept at low temperatures in a temperature-controlled greenhouse or grown in naturally cool environments such as in high mountains. In the laboratory, low-temperature treatment has been adopted for shortening the juvenile phase and promoting flowering [19]. Plants exposed to a constant temperature of 13°C flowered early whereas those at 18°C remained vegetative and did not flower [18, 20]. To summarize, vernalization can promote flowering in this species. However, the genetic basis of low-temperature-induced flowering in *D. nobile* remains poorly understood.

Although a number of genes have been identified in orchids [21, 22], and several datasets of expressed sequence tags (ESTs) from flowering buds of *Orchidaceae* species have been deposited in GenBank, only a few of these genes have been characterized. Examples come to *DOMADS1* and *DOH1* from* Dendrobium *Madame Thong-In which are believed to be involved in the floral transition [23, 24]. Genetic networks underlying the biological features of vernalization in orchids have not yet been established.

In the present study, we created a collection of ESTs from axillary buds of *D. nobile* plants that had been vernalized for different durations. Changes in gene categories over time during the course of vernalization were studied and a group of candidate genes involved in flowering were identified. Based on the EST analysis, we propose a preliminary genetic network for vernalization-induced flowering in *D. nobile*.

## 2. Material and Methods

### 2.1. Plant Material

Plants of *D. nobile* were grown without regulating the photoperiod in a greenhouse in School of Life Science at South China Normal University (China). Adult plants were selected for experimental use. Plants for the low-temperature treatment were moved to an air-conditioned greenhouse with the temperature setting at 15°C during the day and 10°C at night. The temperature regime was maintained over a different number of days (5, 10, 20, and 30 days) and the plants were sampled immediately upon moving to the greenhouse (0 d) and after the set duration (5 d, 10 d, 20 d, and 30 d). Control plants were grown under natural conditions (the average temperature was 26.1°C). A total of 5–7 axillary buds close to the shoot tip of each seedling were harvested at the same time of the day at each sampling to avoid the possible influence of circadian rhythms. Excepting the whole apex, some other subtending tissues including the vascular transition zone and immature leaf bases had to be retained due to the difficulties in sampling. Totally, 70 seedlings were sampled, and the buds from each treatment were pooled. All buds were frozen under liquid nitrogen and stored at –80°C until required.

### 2.2. RNA Extraction and Purification

Total RNA was prepared from the axillary buds from each treatment and control sample. Total RNAs from two different samples were pooled as described in additional file 1 in Supplementary Material available online at doi: 10.1155/2012/757801 for library construction, except for the control one (0 d). RNA was extracted using the RNeasy Plant Mini Kit (QIAGEN, Germany) but replacing the Trizol-based extracting buffer with a CTAB-containing one (2% CATB, 1% PEG4000, 1.4 M NaCl, 1 mM Tris·HCl and 20 mM EDTA, pH 8.2~8.5).

### 2.3. Library Construction

Full-length cDNA libraries were constructed using the Creator SMART cDNA Library Construction Kit (Clontech, USA) according to the manufacturer's instructions. The recombinant pDNR-LIB with *D. nobile* cDNA insertions was transformed into *E. coli* DH5*α* and spread on the LB argar containing chloramphenicol. Monoclones were recovered by random selection.

### 2.4. Sequencing

Plasmids were isolated using the alkaline lysis method and used as starting templates for the subsequent sequencing. Insertions were sequenced from the 5′-end primed by M13 forward-sequencing primer using the Applied Biosystems 3730 DNA Analyzer. Before large-scale sequencing, hundreds of monoclones were selected and sequenced to assess the frequency of both unloaded clones and multiple clones.

### 2.5. EST Analysis

Sequences longer than 100 bp (600 bp on average) were considered for later analysis. Base calling and quality assignment of individual bases were done through Phred (*Q* = 20) [25]. All the clean ESTs were assembled using Phrap (minimatch = 42 and miniscore = 20). The assembled sequences (including contigs and singlets) were submitted to BLASTX for comparison against the nonredundant protein database available at NCBI (http://www.ncbi.nlm.nih.gov/) and the *Arabidopsis* protein database (TAIR10 proteins) at TAIR (http://www.arabidopsis.org/). ESTs from a single library were assembled and underwent BLASTX searches independently. Unigenes from different libraries were considered identical if the best hits in BLASTX matched the same protein.

To confirm the transcriptional change of the identified ESTs, real-time qPCR was performed for candidate ESTs or full-length cDNAs believed to be involved in flowering. Total RNA from each treatment duration was used as starting templates. Primer pairs for each candidate are listed in additional file 2.

For protein comparison, MEGA3 [26] was used to align homologous sequences. Amino acid identities were shown as percentage of conserved amino acids by pair wise comparison of the candidate protein and its corresponding homolog.

### 2.6. Gene Ontology (GO) Annotation

Gene ontology annotations were assigned to each unigene of *D. nobile* according to the best hit in BLASTX against the *Arabidopsis* TAIR10 protein database. Classifications were performed on the GOSlim-plant ontology of biological process. The frequency of each GOSlim-plant term was calculated as follows: (the number of nonredundant mUnigenes annotated by this GOSlim-plant term/total nonredundant annotated mUnigenes) × 100. If a unigene was annotated by several GOSlim-plant terms, it was classified into each of these terms but counted only once for the total count of nonredundant unigenes. GO annotations and GOSlim-plant categories of *Arabidopsis* proteins were retrieved as references from the TAIR website (http://www.arabidopsis.org/).

Overrepresented GO categories were extracted with BiNGO 2.3 [27] using hypergeometric statistics test and Benjamini and Hochberg False Discovery Rate (FDR) correction. GO categories with a corrected *P* value <0.05 were considered overrepresented based on comparisons with the whole annotation of *Arabidopsis*. All these comparisons were performed on the ontology of biological process.

### 2.7. Real-Time Quantitative PCR

Total RNA was extracted from axillary buds of *D. nobile* plants exposed to low temperatures for 0, 5, 10, 20, and 30 days. In most cases, buds from 3 to 5 adult plants were pooled before RNA extraction. The qPCR reactions were run in the ABI PRISM1 7300 (Applied Biosystems) using the following program: hot start at 95°C for 30 seconds followed by 40 cycles of 5 seconds at 95°C and 31 seconds at 62°C. In each 20 *μ*L qPCR reaction, cDNA initially generated from 5 to 7.5 ng total RNA was used as a template. The qPCR reaction also contained gene-specific primer pairs (250 nM each) and 10 *μ*L SYBR Green Premixture (TaKaRa) and 0.4 *μ*L Rox (TaKaRa). A total of 9 genes were assayed and the 18sRNA gene was used as the endogenous control. The primers used are listed in additional file 2. The sample from the 0 d batch was used for calibrating the expression level of each treatment duration. Three technical replicates were used for each combination of genes and the duration of exposure (days). Sterile distilled water instead of the cDNA template was used as the negative control. All the data were analysed using the SDSv1.3.0 software (ABI) based on the 2^−ΔΔCt^ method [28]. The RQ values representing the relative expression level of a given gene under different treatment durations were log 2-transformed in later analyses.

## 3. Results and Discussion

### 3.1. EST Collecting and Sequencing from *D. nobile* Axillary Buds

**Figure 1:**
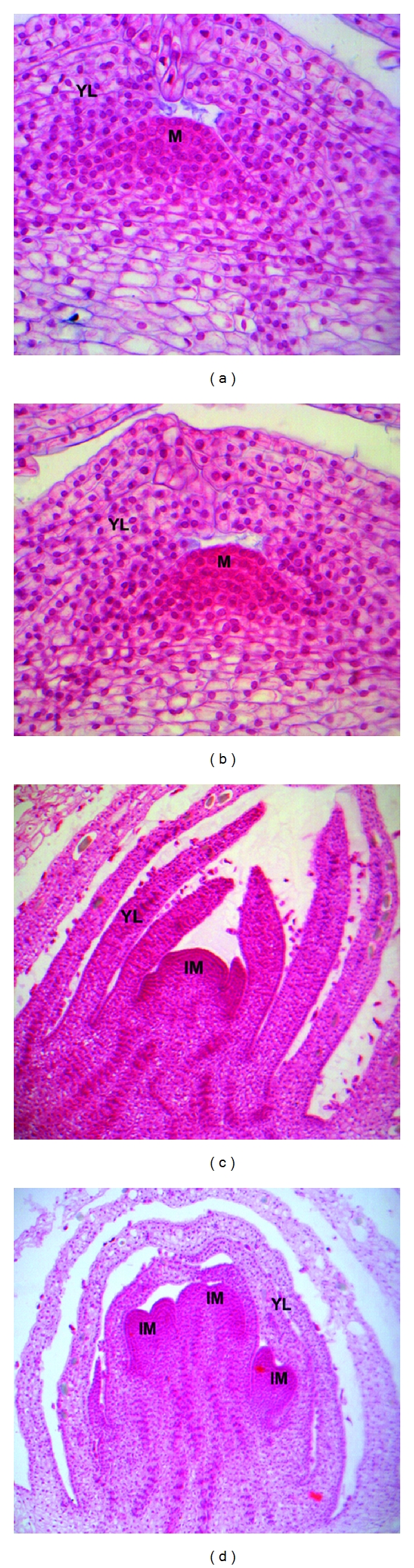
Low-temperature-induced anatomical changes in axillary buds of *D. nobile*. Axillary buds were sampled from seedlings of *D. nobile*, which were vernalized at 15/10°C (day/night) for (a) 0, (b) 10, (c) 15, and (d) 30 days. The buds were first fixed by FAA and stained with Ehrlich's haematoxylin, and then embedded in paraffin to section serially. Anatomical examinations were carried out under microscope with magnification of 40 × 10 (a, b) and 4 × 10 (c, d). Visible changes could be found on meristem tissues of axillary buds after low-temperature exposure, from bulge initiation (b) and development (c), to differentiation of floral primordia (d). M: Meristem; YL: Young Leaf; IM: Inflorescence Meristem.

Low-temperature treatment (artificial vernalization) of *D. nobile* can shorten the vegetative stage and accelerate flowering [19]. When compared with nonvernalized control, 10 days of exposure to low temperature produced a bulge in the node which would develop into several flower buds later, indicating that the transition from the vegetative stage to the flowering stage occurs at that time (Figure 1(b)). Extended low-temperature treatment hastened the development process (Figures 1(c) and 1(d)) and eventually led to the formation of floral organs from the bulged meristem.

To monitor the transcriptional changes during the low-temperature-induced floral transition, three libraries were constructed using axillary buds collected at the end of the five treatment durations (0, 5, 10, 20, and 30 d). The bud samples were pooled, following the scheme shown in additional file 1, to increase the variation in transcripts from different libraries. Another cDNA library constructed previously from 40-day-vernalization buds [29] was also included in the present study (additional file 1). Thus, we obtained four cDNA libraries presenting transcripts from distinct stages of vernalized buds. These libraries made it possible to perform a combined analysis and to detect changes in transcript abundance over time during vernalization.

About 3800 clones from each cDNA library were selected for sequencing, and 15 381 reliable reads were obtained from the four libraries (GenBank accession numbers HO189246–HO204626). After removing the low-quality reads and vector contaminants, 15 017 clean ESTs were left, divided as follows: 3391 from the 0 d library, 3369 from the (5+10)d library, 4112 from the (20+30)d library, and 4145 from the 40 d library (Table 1). The average length of EST was 650 bp, and most fell into the range of 400–800 bp (additional file 3). Reads from different libraries were assembled independently using Phrap, resulting in 2976, 2983, 3491, and 3648 nonredundant Unigenes (including aSinglets and aContigs in Table 1) from the four datasets, respectively. The average percentage of nonredundant EST was 87.3 (and ranged from 84.9 to 88.54), indicating a relatively low redundancy within each library.

The number of the aContigs was probably underestimated in the assembly step, as some pseudounassembled ESTs can match to the same protein in BlastX analysis. We placed such EST sequences into a contig manually and designated as a mContigs (additional file 7). An EST sequence that matched to a given protein exclusively was designated as an mSinglet (additional file 7). After such manual processing, the number of mUnigene (mSinglet +mContig) was lower than that generated from the original assembly (Table 1).

Cutting by *E* value <1*e*-5, more than 77% ESTs on average from each library matched to at least one entry in BlastX searching against the nonredundant protein database at NCBI. Based on the best match, 7802 records were retrieved, some of which were shared by two or more libraries. After removing those repetitive records across libraries, annotated mUnigenes were up to 6375 (Table 1). The number of records unique to each library was as follows: 1305 to the 0 d library, 1275 to the (5+10)d library, 1479 to the (20+30)d library, and 1231 to the 40 d library. The absence of these sequences from other libraries is probably a result of inadequate reading of the sequences from each library. Overlapping analysis between libraries revealed that 4378 mUnigenes were absent in the 0 d library, out of which 28 were shared by the three vernalized libraries (additional file 4). Proteins involved protein synthesis and postprocessing, including several ribosomal proteins, an RNA-binding protein, a subunit of transport SEC1 protein, and an ubiquitin fusion protein, were predominant in this shared gene set.

### 3.2. Functional Annotation and Classification of *D. nobile* Unigenes

**Figure 2:**
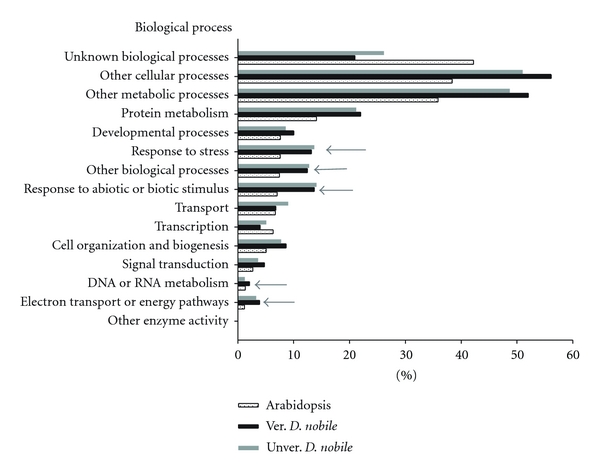
Classifications of unigenes based on GOSlim-plant ontology of biological process. GO terms were assigned to *D. nobile* unigenes according to best hit in BLASTX against *Arabidopsis* protein database (http://www.arabidopsis.org/). Classification was conducted at http://www.arabidopsis.org/tools/bulk/go/index.jsp for unvernalized (grey bars) and vernalized (black bars) *D. nobile* gene sets according to GOSlim-plant annotations on biological process. Spectrums of unigene categories were similar between these two gene sets. Unigenes that grouped into the “unknown biological process” category are 26.17% and 21.01% of the vernalized and unvernalized dataset, respectively. The *Arabidopsis* whole annotation is used as a reference (dotted bars). Gene categories with approximate 2-fold increase in *D. nobile *relative to that in *Arabidopsis* are indicated by arrows.

BLASTX searching showed that 78%, 72%, 70%, and 42% aUnigenes from the four libraries matched at least one record in the TAIR10 protein database, respectively, (*E* value <1*e*-7). By manually removing the redundant records, the number of annotated unigenes, namely, mUnigenes, came to 1599, 1553, 1756, and 1163 for the four libraries, respectively. GO terms were retrieved for approximately 89% of these Unigenes (Table 1). To develop a clear, functional classification for the annotated mUnigenes, the initial GO terms were first converted to GOSlim-plant terms and the frequency of each GOSlim-plant term was calculated as described in Section 2. The overall pattern of distribution of Unigenes in *D. nobile* was similar to that in *Arabidopsis* (Figure 2). However, the categories “response to stress,” “response to biotic and abiotic stimulus,” and “electron transport and energy pathway” were more pronounced in *D. nobile*. Low-temperature treatment seems to lead to small changes in *D. nobile*. Vernalization-induced increase in the frequency of categories “DNA and RNA metabolism” and “signal transduction” was less than 2 folds, whereas no obvious changes were observed in the frequency of “cell organization and biogenesis,” “development process,” “electron transport or energy pathways,” and “protein metabolism.” These results implied that *D. nobile* would maintain a constant transcription level during vernalization. Similar observations were previously reported in wheat, in which the transcript accumulation in crown tissue was lower in winter varieties than that in spring varieties during cold acclimation [30].

### 3.3. Functional Enrichment in Vernalized *D. nobile*

To identify the overrepresented gene categories, we performed a functional enrichment analysis using BinGO software [27]. Referring to the whole annotation of *Arabidopsis* transcriptome (TAIR10), the gene category “response to stress” was overrepresented in the axillary buds, considering either before or after exposure to low temperature. This phenomena was likely linked to cold, high salinity, and heat stresses (data no shown), and the cold-induced responses may be the major theme because cold-responsive genes were increased dramatically in the total number with time during the whole vernalization process. Among the cold-responsive gene set, a homolog of *Arabidopsis AGL19*, a key regulator in vernalization-associated flowering, was presented, and transcript quantification confirmed that the initial induction of this *AGL19* homolog occurred just after 5 days of vernalization (Figure 5), implying that transition to the flowering phase would be initiated early by vernalization. Forty days of vernalization is probably in relation to cold adaptation, as two cold-acclimation-related genes, one similar to *Arabidopsis CAX1* and the other to *ATRZ-1A* [31], were induced at this stage. These observations suggested a link between the initiation of floral transition and the cold acclimation in *D. nobile*. Besides, responses to oxidative stress, zinc ion, and light stimulus were also remarkable, but they were vernalization-stage-specific and could not be retained when low-temperature exposure was extended over a longer duration (Table 2). Genes involved in metabolism of carbon, nucleotide, and amino acid were also enriched before vernalization and in the early stage of low-temperature treatment (Table 2). Differing from the changes on stress-related responses and metabolism status, regulations of translation, protein synthesis, and chromatin assembly became activated after a long-term of vernalization, indicating posttranscriptional regulation might have important roles in low-temperature-induced responses in *D. nobile*.

The “flower development” subcategory was not overrepresented as expected, which led to a speculation that transition to the flowering stage would not occur, but we found that the frequency of this subcategory increased gradually with time during the vernalization process, and many homologs involved in various pathways promoting the floral transition and flowering development were induced after the low-temperature treatment. This suggested that the transition may have begun although it was not detected at this stage, which is reasonable because exposure to low temperature for 30–35 days is enough to promote flowering in *D. nobile*.

### 3.4. Time-Course Change in Biological Processes in *D. nobile* during Vernalization

**Figure 3:**
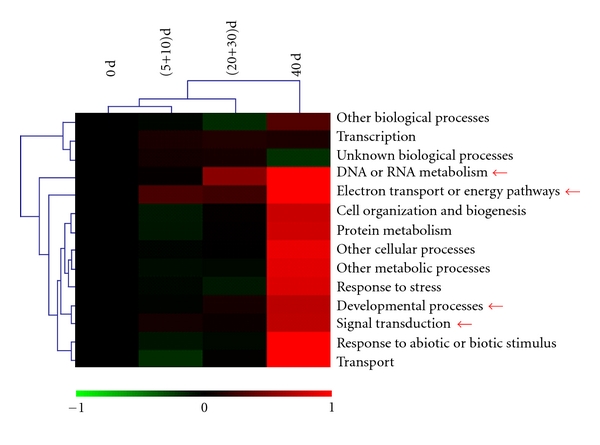
Time course profile of gene categories (GOSlim-plant terms). Hierarchical clustering analysis was performed to monitor the time-course profiles of gene categories basing on the fold changes relative to 0 d. The arrows indicate the gene categories that were initially induced after short term vernalization and activated after long-term vernalization.

The increasing number of overrepresented categories implies that the overall transcription is activated as vernalization proceeds (Table 2). Clustering analysis revealed that some GOSlim-plant categories had followed such a trend, including “DNA and RNA metabolism,” “development processes,” “electron transport and energy pathways,” and “signal transduction” (Figure 3). Besides, the 0 d set was initially grouped with the (5+10) dataset, indicating that the transcript profile at the initial stage of vernalization was similar to that during vegetative development. Midway through vernalization (the (20+30) dataset), genes involved in “DNA and RNA metabolism,” “development process,” and “electron transport and energy pathways” were induced (Figure 3). The final stage (40 days of vernalization) was clearly distinct and showed greater and more extensive induction of genes (Figure 3). Together, these results suggest two types of transcriptional regulation during vernalization in *D. nobile*, cellular homeostasis at the early stage and systematic activation at the late stage.

### 3.5. Homologs of Cereal Vernalization-Responsive Genes

Based on the functional prediction and enrichment analysis, we identified 59 *D. nobile* genes whose *Arabidopsis* homologs are annotated involving the flowering development or are related to development of floral organs (Table 3 shows 13 genes as examples). We initially focused on those putative orthologs and functional equivalents that have been reported to be involved in vernalization or those relevant pathways.

Cereal *VRN1*, together with *VRN2*, *VRT2*, and *VRN3*, forms the major regulation pathway that controls vernalization-induced flowering in temperate cereals [1, 2], in which *VRN1* and *VRN3* serve as flowering activators while *VRN2* and *VRT2* as repressors. TaVRT2 represses transcription of the wheat vernalization gene *TaVRN1*. Cereal VRN2 is similar to the *Arabidopsis* CO and CO-like proteins, containing a CONSTANS, CONSTANS-like, and TOC (CCT) domain [32]. It acts as a negative regulator in flowering and is suppressed after vernalization. Heterologous expression of *TaVRN2* can delay flowering in transgenic *Arabidopsis* [33]. However, no orthologs of the *TaVRN2* locus have been found in *Brassicaceae* [15], and the origin and evolution of *VRN2* in wheat and other monocots remain a mystery. In the present study, we did not identify the *VRN2* ortholog in *D. nobile*, possibly duo to limits of techniques.

However, several EST sequences similar to *VRN2*'s interactor, *VRT2*, were identified. Cereal *VRT2* is homologous to *Arabidopsis SVP* and *AGL24* and possibly downregulated by vernalization [34, 35]. Transcription of the *D. nobile VRT2*-like homolog was not repressed but activated by low temperature (Figure 5), which is different from what is observed in wheat *VRT2* but mimics the behaviour of *AGL24* in *Arabidopsis *[36]. The role of this *D. nobile VRT2-like* gene is not clear, and further studies are necessary to ascertain its involvment in vernalization-induced flowering.

**Figure 4:**
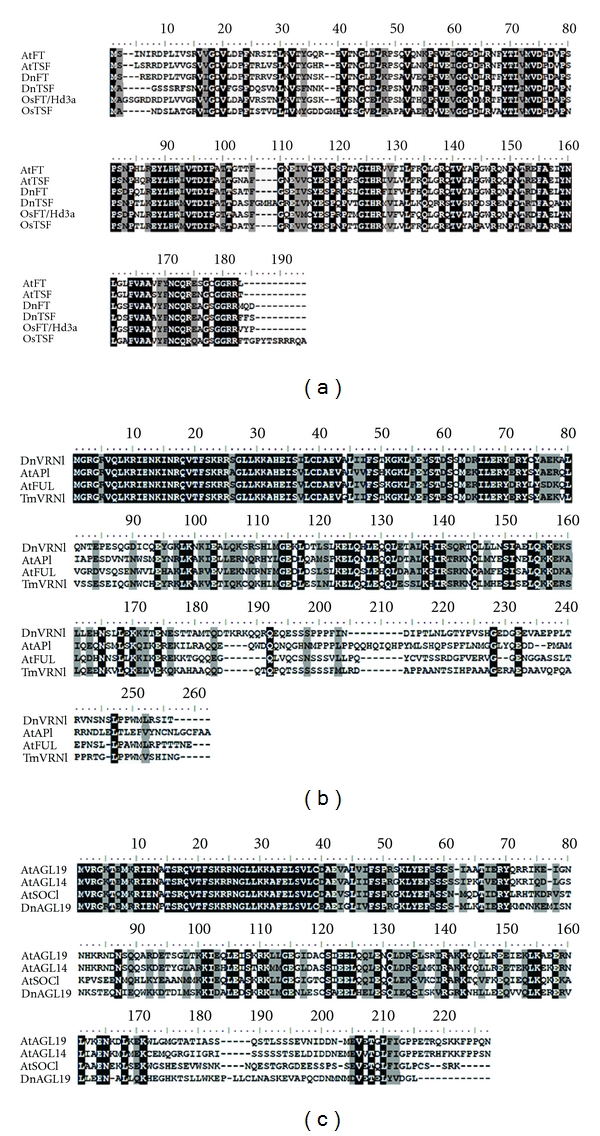
Alignment of FT, VRN1, and AGL19 homologous proteins. Phylogentic analyses are shown in additional file 6. (a) DnFT and DnTSF are aligned with FT homologs from* Arabidopsis* (At) and rice (Os). Phylogenetic analysis indicates that DnTSF is closer to OsTSF while DnFT is to OsFT. (b) DnVRN1 is compared with *Arabidopsis* AP1, FUL and wheat VRN1. (c) DnAGL19 is aligned to *Arabidopsis* AGL19, AGL14, and SOC1. Distance (branch length) between DnAGL19 and AtAGL19 is less than that between it and AtSOC1. Identical sites are shaded in black, while those similar sites are in grey.

**Figure 5:**
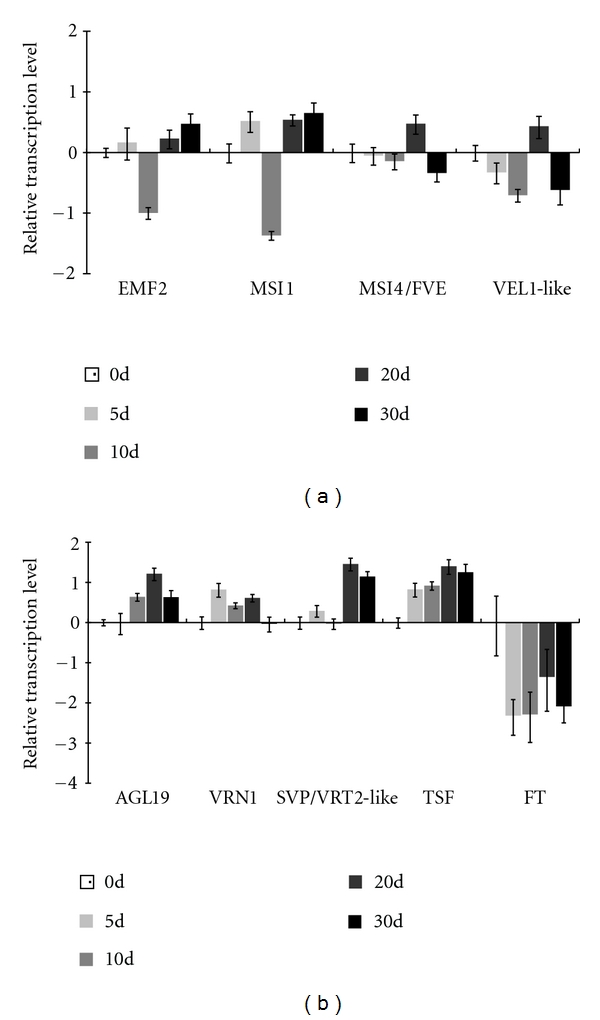
Time-course transcript profiles of selected flowering-relevant genes during the vernalization process. Relative expression levels of each selected genes after exposing at 15°C/10°C (day/night) for 0, 5, 10, 20, and 30 days are shown. Total RNA were isolated and were reverse transcribed to cDNA that served as templates in real-time qPCR assays. RQ values of each gene relative to 0 d-bud samples were log 2-transformed (*y*-axis) and plotted against the time periods (*x*-axis). The positive and negative values indicate the vernalization-induced activation and repression, respectively. The error bars are calculated based on the (RQMin and RQMax) confidence interval (95%).

**Figure 6:**
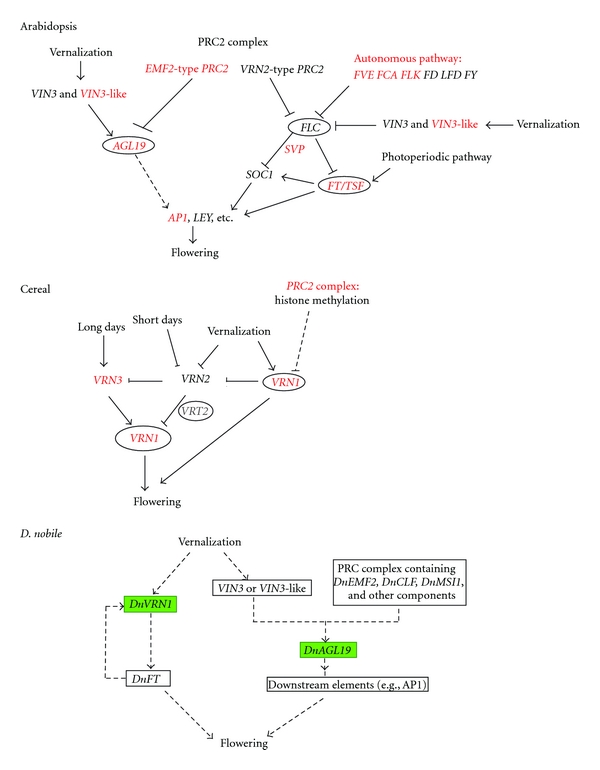
Predicted vernalization regulation networks in *D. nobile*. By comparative analysis with *Arabidopsis* and temperate cereal, we found many *D. nobile* unigenes induced by vernalization are homologs or functional equivalents of the flowering regulators. These homologs (highlighted in red) involve two vernalization pathways, one is the *Arabidopsis AGL19* pathway and the other is the cereal *VRN1* pathway. FLC homolog cannot be identified from *D. nobile* and this indicates that FLC mediated regulation might be absent or be repressed during vernalization process. Networks to regulate flowering by the vernalization, similar to those in *Arabidopsis* and temperate cereal, are proposed in *D. nobile*. It should be noted that this network must be confirmed by more extensive studies basing on more solid evidences. The network chats of *Arabidopsis* and temperate cereal are adopted from the article by Alexandre and Hennig [1], or are summarized according to the descriptions in articles by Higgins et al. [8], Kim et al. [2], and Trevaskis et al. [9, 10].

Vernalization-associated repression of *VRN2* transcription in some cereals is probably linked to the induction of *VRN1*, a flowering activator [37]. Cereal *VRN1* is the ortholog of *Arabidopsis AP1*, an A-class gene that functions in identifying floral meristem [38]. In temperate cereals, it has more functions such as cold signal transmission during vernalization [2]. The gene is initially transcribed at low levels and induced by vernalization under both long- and short-day conditions [39, 40]. Activation of this gene can subsequently induce *FT/VRN3* expression in leaves, which makes plants be capable of flowering (Figure 6). Therefore, *VRN1* has been suggested to be the primary regulator of vernalization-mediated floral transition in temperate cereals. Orthologs of *VRN1* have been isolated from many monocots, including wheat, barley, oat, and rye [40–42]. A *D. nobile* homolog of *VRN1* was also identified in this study. This gene codes for a protein consisting of 247 amino acids and has a typical MADS-box domain located at the 5′-end (Figure 4). Pair wise comparisons indicated that the deduced peptide sequence had 48.5%, 53.8%, and 61.5% overall identities with AtAP1 (AGL7), AtFUL (AGL8), and TmVRN1 (AAZ76882.1) (additional file 6), respectively, and we designated it, *DnVRN1*, to indicate its close relation to cereal *VRN1*. Transcription of this gene in buds was initially induced after vernalization for 5 days, remained constant thereafter, and decreased slightly after long-term vernalization (Figure 5). This pattern of expression was somewhat similar to that of *VRN1* in wheat and barley [42–44].

In addition to *VRN1*, cold-induced reduction of *VRN2* is also linked, at least in barley, to PPD1-dependent induction of *VRN3*, an ortholog of *Arabidopsis FT* [11, 16]. *FT/VRN3* may be expressed in leaves and the protein is then translocated to the shoot apex, where it triggers the transition to flowering [45]. Two *VRN3-like* transcripts were identified from the axillary buds of *D. nobile*. The first one codes for a protein of 178 amino acids similar to *Arabidopsis FT* and *TSF* with identities of 57.3% and 56.7%, respectively, (Figure 4 and additional file 6). When compared with *OsFT *(rice Hd3a, BAB61030), *OsTSF *(NP_001046372), and *Triticum aestivum VRN3* (TABK32208.1), the amino acid identity rose to 59.2%, 62.4%, and 55.6%, respectively. Phylogenetic analysis also indicated it was closer to *OsTSF* than to *OsFT* (additional file 6). Thus, we named this gene *DnTSF*. Only one EST of this gene was observed in the (5+10)d library (Table 3), but real-time qPCR assay showed that it was induced in buds throughout the entire duration of vernalization (Figure 5). Another *VRN3*-like transcript was identified from the 40 d library (Table 3). The full-length cDNA of this transcript was also indentified (Figure 4). Phylogenetic analysis revealed that it was close to *OsFT*, so we designate it *DnFT* (additional file 6). The expression level of *DnFT* in buds remained at very low level under vernalization (Figure 5), unlike that of *DnTSF*. In leaves, however, the expression of *DnFT* was lower before vernalization (Liang, S, unpublished data) and was induced to a higher level by vernalization (Li, R-H, unpublished data). Together, these results led to a speculation that vernalization would induce *DnFT* in leaves, and the protein then be translocated to the shoot meristem to serve as florigen. Thus, the expression and the function of *DnFT* may be similar to their orthologs in *Arabidopsis*, wheat, and barley [46]. *DnTSF*, on the other hand, is probably regulated by means distinct from those in relation to *DnFT* and may form a part of another pathway as that being proposed in *Arabidopsis* [47].

To summarize, we identified *D. nobile* homologs of cereal vernalization-responsive genes, including two essential components, *DnVRN1* and *DnFT*, and the *VRT2-like* gene (Figure 6). Vernalization induced the transcription of *DnVRN1* in axillary buds where the inflorescence would emerge later (Figure 5), suggesting that *DnVRN1*, like its homologous gene in cereal [42], would involve in the transition to flowering. Another component, DnFT protein, was probably synthesized in leaves and translocated to the shoot meristem after vernalization where the feedback loop between *DnVRN1* and *DnFT* is established. The close homolog of *DnFT*, namely, *DnTSF*, probably regulated flowering in *D. nobile* in a way distinct from that followed by *DnFT*. Our observations pointed to a *VRN1*-mediated pathway in *D. nobile* which evolutionarily conserved between this species and temperate cereals. However, this possibility needs to be confirmed by further studies.

### 3.6. Absence or Repression of *FLC*-Dependent Pathway in Vernalized *D. nobile*

*FLC* is considered as the central component in vernalization-induced floral transition in *Arabidopsis *[2]. However, the *D. nobile FLC* homolog was not found in our dataset, even in the 0 d samples being expected to contain high levels of *FLC* transcripts. One possible explanation for the absence of *FLC* transcript is that the orthologous gene of *Arabidopsis FLC* did not exist in *D. nobile* genome *per se*, like cases in some temperate cereals [37].

However, an alternative explanation that *D. noble FLC* homolog was repressed by vernalization is also reasonable. Some transcripts involved in *FLC*-dependent vernalization responses were found in vernalized *D. nobile* (Table 3), including two positive regulators of *FLC,* homologs of *VIP4* and *VIP5* [48, 49]; an interaction partner of *FLC, SVP *[50]; and some components relevant to *FLC* chromatin histone modification, such as *CLF*, *MSI1*, *UBC1*, *UBC2*, *HTA9*, and *HUB1* [2]. These observations, together with the absence of *FLC* transcript, implied that an *FLC* homolog would exist in *D. nobile* but would be in fact repressed by activation of autonomous pathway in axillary buds (indicated by the higher level of transcription of *FVE* after exposure to low-temperature, Table 3 and Figure 5) or by increasing enrichments of H3K27 methylation on the *FLC* locus through activities of polycomb repressive complex 2 (PRC2, Table 3) [1, 3]. This repression consequently led to lower level of *FLC* expression and made it difficult to identify its transcript through the experimental techniques used in present study.

### 3.7. Autonomous Pathway Homologs

An autonomous pathway represses the transcription of *FLC* in *Arabidopsis *[51]. Components of this pathway, including *FCA*, *FVE* (aka *MSI4*), and *FLK*, were also identified from *D. nobile *buds. *D. nobile FVE* homolog was probably expressed constitutively in buds of *D. nobile* (Figure 5), indicating that it was not affected by vernalization. The level of *D. nobile FCA* transcript may decrease during the course of vernalization (Table 3), but the effect of this decrease could not be assessed in the present study and should be confirmed by further experiments.

### 3.8. Homologs Involved in Chromatin Histone Methylation

Polycomb repressive complex 2 contributes to the maintenance of chromatin H3K27 methylation in plants. The VRN2-containing PRC2 complex (not the equivalent of cereal *VRN2*) consistently suppresses the expression of *FLC* in *Arabidopsis*. However, *VRN2* homologs were not found in rice [8], nor could we find in the *D. nobile* EST collection, which suggested that this gene, as well as the VRN2-containing PRC2 complex, may be absent in this *Orchidaceae* species. On the other hand, the EMF2-containg PRC2 complex probably play a role in the response to vernalization in *D. nobile*. The *D. nobile EMF2* homolog and two other genes, *CLF* and *MSI1,* were transcribed in buds (Figure 4), and vernalization-induced transcription of these proteins may be related to H3K27 methylation of some gene loci, possibly, including the *AGL19* locus, but not to that of *FLC* [5].

### 3.9. *AGL19* Homolog and *AGL19*-Mediated *FLC*-Independent Pathway

Two *FLC*-independent pathways, one mediated by *AGL19* and the other by *AGL24*, can promote the transition to flowering in *Arabidopsis* [1, 2]. The absence of *FLC* transcripts in *D. nobile* implied that *FLC*-independent vernalization pathway(s) would play important roles in response to vernalization. It is noteworthy that a vernalization-induced transcript (Table 3, Figure 5) had 52.7% and 48.8% similarities at the level of amino acids to *Arabidopsis AGL19* and *SOC1,* respectively, (Figure 4, additional file 6). Despite such close similarities, the deduced peptide of this transcript diverged more from AtSOC1 than AtAGL19, suggesting that the peptide is closer to AGL19. We, therefore, designated it *DnAGL19*. Transcription of *DnAGL19* was activated after 20 days of vernalization (Figure 5), a situation somewhat similar to that of its homolog in *Arabidopsis* [5]. On the other hand, transcription of *DnAGL19* was probably related to the EMF2-containg PRC2 complex (Figure 6). Expression of the *D. nobile EMF2* and *MSI1* homolog was suppressed early on during vernalization (10 days) and increased slightly thereafter (Figure 5). Repression of these genes early on would be related to induction of *DnAGL19* (Figure 5) and implied that the EMF2-complex was involved in the regulation of *DnAGL19* by histone methylation. A long period of vernalization may help in restoring the transcription levels of *EMF2* and* MSI1,* which may in turn lead to the re-establishment of H3K27m3 at some gene loci. In brief, a *DnAGL19*-involved regulation network, similar to the *Arabidopsis AGL19* pathway, may be conserved in the monocotic *D. nobile*. However, more experimental studies are needed to confirm this possibility.

## 4. Conclusions

From a collection of ESTs derived from buds of vernalized *D. nobile* plants, 9616 nonredundant unigene sequences were identified, of which approximately 64% matched at least one record in the nonredundant protein database at NCBI or in the *Arabidopsis* TAIR10 protein dataset. GO annotations based on ontology of biological process were assigned to each unigene according to the best match in BLASTX. Three libraries, corresponding to critical lengths (number of days, d) of vernalization ((5+10)d, (20+30)d, and 40 d), provided insights into changes in gene categories. As expected, genes including those in the “response to cold” category were significantly predominant during vernalization, and genes that control flowering time were initially induced early on. These results indicate that vernalization causes dynamic changes in the transcriptome and induces flowering in *D. nobile*, which may crosstalk with cold acclimation.

The absence of *FLC* in the EST collection indicated that, as in other monocots, an *FLC*-dependent pathway may not exist in *D. nobile*. However, this requires confirmation by extensive evidences. Close homologs of vernalization-responsive genes of temperate cereals, including the *DnVRN1* and *DnFT*, as well as a homolog similar to *Arabidopsis AGL19*, were identified, pointing to the presence of similar regulation networks in vernalization-induced transition to the flowering in *D. nobile* (Figure 6).

Information on preliminary networks regulating the transition in *D. nobile* (Figure 6) will facilitate further characterization of the key players in vernalization pathways in *Orchidaceae*. Our findings pave the way to greater understanding of the vernalization network in *D. nobile*, which will provide novel insights not only on the mechanism that controls flowering time but also on the genetic basis underlying the evolutionary adaptation to cold in *Orchidaceae*. Finally, exploring the genes that control flowering time will be hoped to benefit in molecular breeding of *D. nobile* for commercially successful cultivars in future.

## Supplementary Material

(1) A scheme of sample pooling for library construction. (2) Primer pairs used in real-time qPCR assays for selected flowering-relevant genes. (3) Distributions of EST size of each library. (4) Venn diagram and a list of ESTs with possible roles in vernalization response. (5) Expression of selected genes in buds of D. *nobile* in response to vernalization treatment were assayed by semi-quantitative RT-PCR. Primer pairs were also listed. (6) Phylogenetic analysis for homologs of DnFT, DnVRN1 and DnAGL19 using MegAlign software embedded in the Lasergene package. Divergent distances are also shown. (7) A chart description of EST assembly.

## Figures and Tables

**Table 1:** Summary of the four libraries.

Library name	0 d	(5+10)d	(20+30)d	40 d	Sum**
Total EST	3391	3369	4112	4145	15017
a*Singlet	2704 (79.74%)	2736 (81.21%)	3166 (77%)	3310 (79.86%)	
aContig	272	247	325	338	
aUnigene	2976 (87.76%)	2983 (88.54%)	3491 (84.9%)	3648 (88%)	
Mean length of EST (bp)	654	615	588	745	

BlastX against nonredundance protein database (Nr) at NCBI					

No hit aUnigene	429	565	770	1477	3241
Hit aUnigene	2547	2418	2721	2171	
m*Unigene^§^	2426	2520	2985	3112	
mSinglet	2016	2145	2539	2657	
mContig	410	375	446	455	

mUnigene^§§^ (Nonredundant across libraries)		6375			

mSinglet		5290 (82.98%)			
mContig		1085 (17.02%)			
Total mUnigene		9616 (sum of 6375 and 3241)			

Blast X against TAIR9 protein datasets at TAIR					

No hit aUnigene	651	812	1032	2087	
Hit aUnigene	2325 (78%)	2171 (72%)	2459 (70%)	1561 (42%)	
mUnigene^§^	1599	1553	1756	1163	
GO annotated mUnigene^§^	1433	1403	1596	997	

*“a” is the first letter of “assembly,” indicating the sequence is generated from assembly using Phrap.

“m” is that of “manual,” indicating the sequence is generated from manually removing the redundancies.

**This column lists simple sum of the 4 libraries.

^§^ and ^§§^ The nonredundant Unigene in the row marked “^§^” are nonredundant within a library but maybe redundant across libraries, while those marked “^§§^” are nonredundant across libraries.

**Table 2:** Significantly over-/under-represented GO identifiers specifically to a stage of vernalizaiton (corrected *P* value <1*e*-05).

	Overrepresented	Under-represented
0 d	Response to stimulus	Gene expression
	Response to zinc ion	Translational elongation
	Metabolism process	
	Purine ribonucleotide metabolic process	
	Methionine metabolic process	
(5+10)d	Metabolism process	Gene expression
	Carbon utilization	Translational elongation
	Metabolic compound salvage	
(20+30)d	Localization	Gene expression
	Protein transport	Translational elongation
	Response to stimulus	
	Denfense response to bacterium	
	Response to light stimulus	
40 d	Gene expression	*None*
	Translation	
	Cellular component organization and biogenesis	
	Ribosome biogenesis	
	Nucleosome assembly	

**Table 3:** Thirteen putative genes of *D. nobile* involved in floral induction.

Putative gene	*Arabidopsis* homologue	EST number			
		0 d	(5+10)d	(20+30)d	40 d
*DnVRN1*	*AP1, cereal VRN1*	0	4	0	0
*DnTSF*	*FT, TSF*	0	1	0	0
*DnFT*	*FT, TSF*	0	0	0	1
*CO-like*	*CO-like 4*	5	0	2	0
*DnAGL19*	*AGL19, SOC1*	0	2	1	0
*DnEMF2*	*EMF2*	0	0	0	1
*DnMSI1*	*MSI1*	1	0	0	1
*DnCLF*	*CLF*	0	0	0	2
*FCA*	*FCA*	1	0	0	0
*FVE*	*FVE*	1	1	1	0
*FLK*	*FLK*	0	1	0	0
*VRT2-like*	*SVP, AGL24, HvVRT2*	2	2	1	0
*VIP-like*	*VIP4*	0	1	0	0

## Abbreviations

*D. nobile*: *Dendrobium nobile*
VRN1: VERNALIZATION 1
AGL9: AGAMOUS-LIKE 19
FLC: Flowering Locus C
VIN3: VERNALIZATION INSENSITIVE 3
VEL: VERNALIZATION 5/VIN3-LIKE
PRC2: Polycomb Repression Complex 2
FT: Flowering Locus T
SOC1: Suppressor of Overexpression of CO 1
H3K27m3: Histone H3 Lys 27 (H3K27) trimethylation
CLF: Curly Leaf
MSI1: Multicopy Supressor of IRA 1
MAF2: MADS AFFECTING FLOWERING 2
VIP4: Vernalization Independence 4
SEC1: Similar to Electron Career 1
CAX1: Cation Exchanger 1
CO: Constans
COL: Constans Like
FUL: FRUITFUL
AP1: Apetala 1
TSF: Twin Sister of FT
SVP: Short Vegetative Phase
UBC: Ubiquitin Conjugating enzyme
HUB: Histone Monoubiquitination
FLK: Flowering Locus KH Domain
EMF2: Embryonic Flower 2.
