# Supplementary material for: Transcriptional Regulations on the Low-Temperature-Induced Floral Transition in an Orchidaceae Species, Dendrobium nobile: An Expressed Sequence Tags Analysis
Source: Comp Funct Genomics. 2012 Apr 9;2012:757801. doi: 10.1155/2012/757801 (PMC3328899; doi:10.1155/2012/757801)

Additional file 1: Scheme of library construction

Library ID	Vernalization time (days)	Stage
0d	0	Vegetative development
(5+10)d	5, 10	Early stage of vernalization
(20+30)d	20, 30	Middle stage of vernalization
40d	40	Late stage of vernalization

Additional file 2: Primers used in real-time qPCR

Name	Primer sequence (5'→3')	Primer length	Amplicon length (bp)
AGL19	CTCTGCTGTGCCTAAATGCT	20mer	114
	AACTGTTGGGTTCTTCCTCC	20mer	
TSF	TCAACTGTCAACGGGAGGC	19mer	101
	GCTATAAGATTAGATAAACAAGGTGCTG	28mer	
FT	CTCAGCAGTGGTGGAGCAGC	20mer	105
	GGATCACTTGGACTTGGAGCAT	22mer	
VRN1	AAGCGGAAACAACAAAGACAG	21mer	144
	CACCCGAGTAAGAGGAGGC	19mer	
VEL1	GCTGACAATGCCCCTGAA	18mer	152
	ACTGATTTTACCATTCTTCTGTGATAC	27mer	
SVP	GCCAGCTGAGAAATATGAGAGGAGATGA	29mer	220
	TTTCCCATTTTTGACATTTCCACGAC	26mer	
EMF2	TGCACCCTAGTACATTGAAGTCT	23mer	198
	TAATAATGTGGGGTAATGTTGATG	24mer	
MSI1	CTTCAGATATAGCAGATGGCAGAGA	23mer	118
	GCACCATCAATTATCCCAAGAAG	23mer	
MSI4/FVE	GCGGGGCATAGAGATAAAGTGGTTG	25mer	199
	GCGCAGGTGAGGATATGGGACTT	23mer	
18s rRNA	GGTTCGCTGCTCGTGACTCT	20mer	161
	CAGGCACCGCTTATTTTTACA	21mer	

Additional file 3:

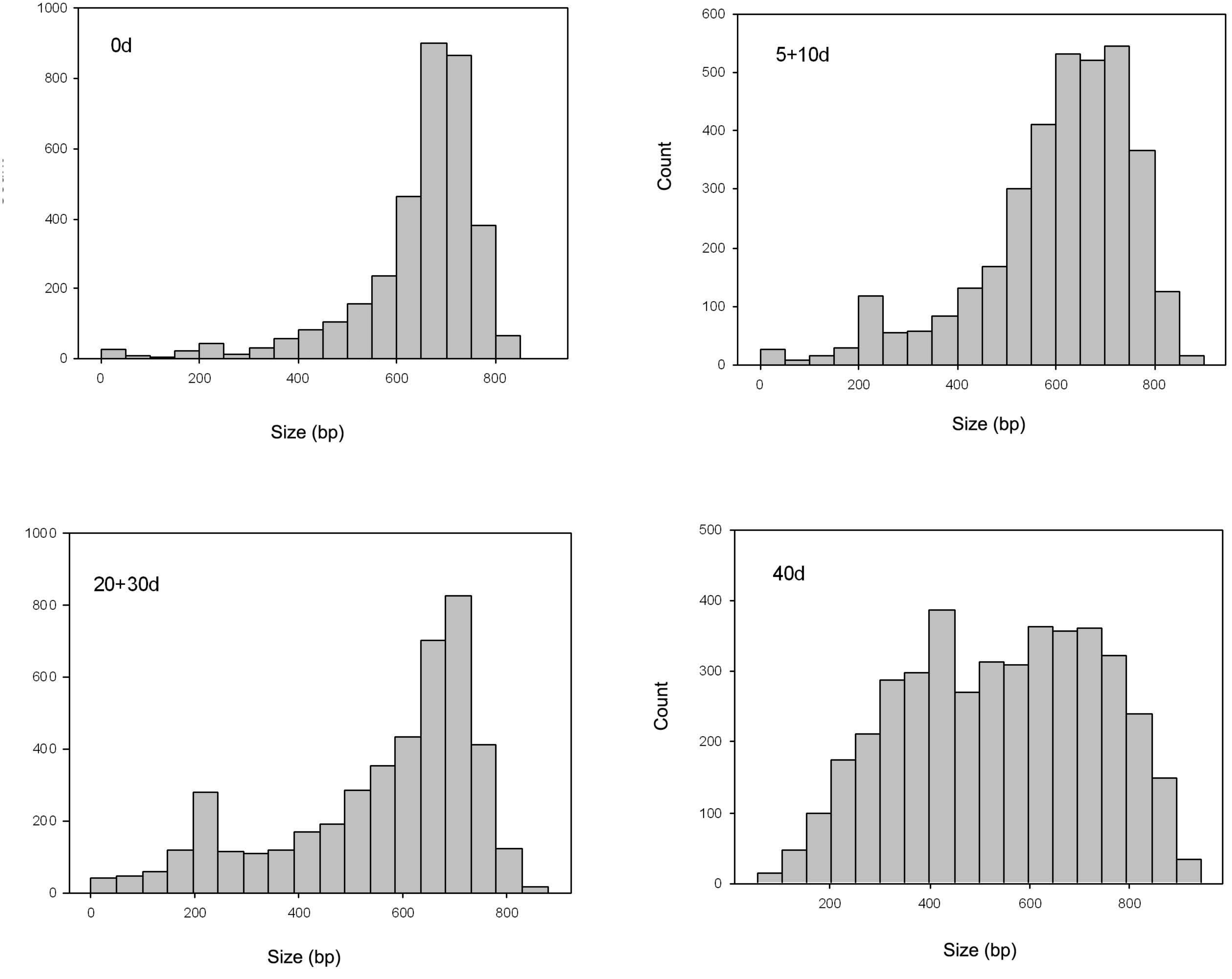

Distribution of EST size

Additional file 4: Venn diagram and list of unigenes presenting in vernalized libraries (5+10d-, 20+30d- and 40d-library).

A total of 4378 unigenes were absent in 0d-library and therefore were assigned as putative vernalization-related unigenes. Of these unigenes, 1561, 1796 and 1442 were from Library_(5+10)d, Library_(20+30)d and Library_40d, respectively, while 28 were shared by three libraries.

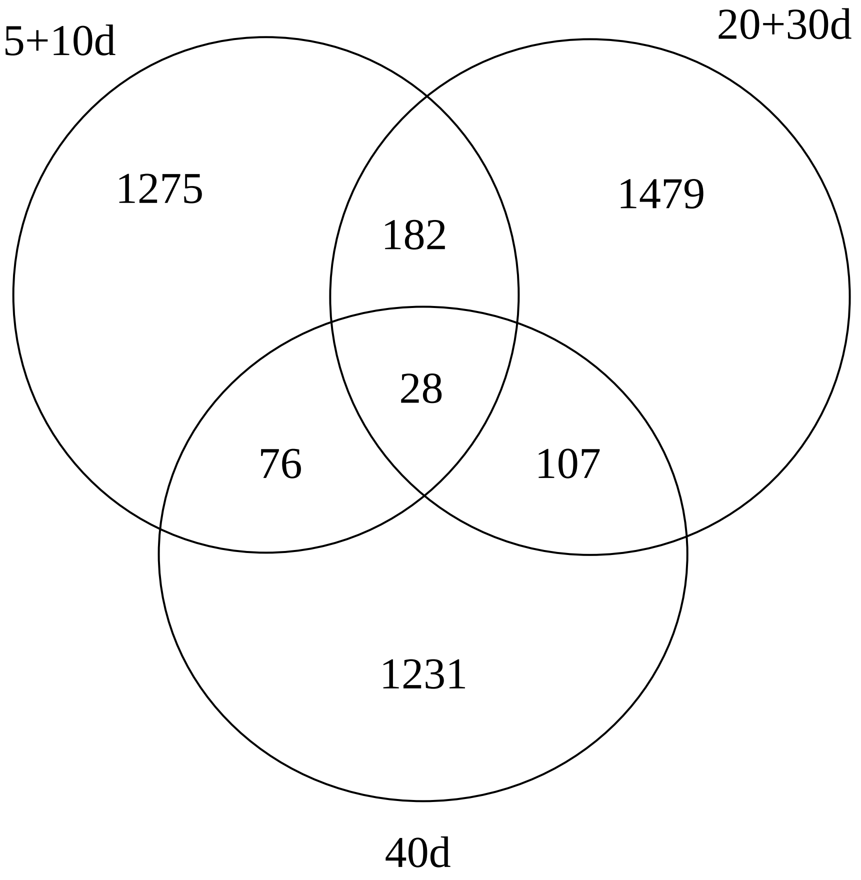

Twenty-eight Unigene shared by the (5+10)d-, (20+30)d- and 40d-library

Genbank Accession No. of homologous gene	E-value	Description	Frequency (‰)		
			5+10d	20+30d	40d
gi|115446513|ref|NP_001047036.1|	4.00E-52	Os02g0534800 [Oryza sativa (japonica cultivar-group)]	0.296824	0.243191	0.482509
gi|115456499|ref|NP_001051850.1|	5.00E-28	Os03g0840900 [Oryza sativa (japonica cultivar-group)]	0.593648	0.243191	0.241255
gi|116309951|emb|CAH66982.1|	6.00E-48	H0714H04.9 [Oryza sativa (indica cultivar-group)]	0.593648	0.243191	0.241255
gi|118482417|gb|ABK93131.1|	9.00E-28	unknown [Populus trichocarpa]	0.296824	0.243191	0.241255
gi|145408430|ref|YP_001152053.1|	2.00E-08	ORF47a [Pinus koraiensis]	0.296824	0.243191	0.482509
gi|147781237|emb|CAN65143.1|	3.00E-67	hypothetical protein [Vitis vinifera]	0.296824	0.243191	0.723764
gi|147783026|emb|CAN65596.1|	6.00E-61	hypothetical protein [Vitis vinifera]	0.296824	0.243191	0.241255
gi|149390985|gb|ABR25510.1|	2.00E-49	tubulin alpha-1 chain [Oryza sativa (indica cultivar-group)]	0.296824	0.243191	0.241255
gi|15224284|ref|NP_181874.1|	1.00E-29	60S ribosomal protein L38 (RPL38A) [Arabidopsis thaliana]	0.296824	0.729572	0.723764
gi|15234793|ref|NP_194222.1|	4.00E-21	protein transport protein SEC61 gamma subunit, putative [Arabidopsis thaliana]	0.296824	0.243191	0.482509
gi|157336898|emb|CAO71583.1|	1.00E-41	unnamed protein product [Vitis vinifera]	0.296824	0.243191	0.241255
gi|157338896|emb|CAO42247.1|	1.00E-26	unnamed protein product [Vitis vinifera]	0.296824	0.486381	0.241255
gi|157342327|emb|CAO64440.1|	6.00E-36	unnamed protein product [Vitis vinifera]	0.296824	0.243191	0.965018
gi|157342485|emb|CAO65197.1|	4.00E-16	unnamed protein product [Vitis vinifera]	0.296824	0.243191	0.241255
gi|157345765|emb|CAO15063.1|	7.00E-90	unnamed protein product [Vitis vinifera]	0.296824	0.243191	0.241255
gi|157354559|emb|CAO47185.1|	6.00E-14	unnamed protein product [Vitis vinifera]	0.296824	0.243191	0.723764
gi|157358359|emb|CAO66019.1|	9.00E-33	unnamed protein product [Vitis vinifera]	1.48412	0.486381	0.482509
gi|166245064|dbj|BAG06616.1|	5.00E-44	ribosomal protein S19 [Dendrobium sp. JLB71]	0.296824	0.243191	0.241255
gi|16930757|gb|AAL32038.1|AF439 279_1	2.00E-31	early light-induced protein-like protein [Retama raetam]	0.890472	0.243191	0.241255
gi|21593543|gb|AAM65510.1|	9.00E-98	60S ribosomal protein L4-B (L1) [Arabidopsis thaliana]	0.593648	0.243191	0.241255
gi|29565662|ref|NP_817244.1|	4.00E-09	ORF45d [Pinus koraiensis]	0.296824	0.486381	0.482509
gi|3122800|sp|O22647|RS16_FRIA G	2.00E-65	40S ribosomal protein S16 ribosomal protein S16 [Fritillaria agrestis]	0.593648	0.729572	0.482509
gi|556367|gb|AAA50310.1|	7.00E-66	light-harvesting chlorophyll a/b-binding protein	0.296824	0.243191	0.482509
gi|55775689|gb|AAV65112.1|	2.00E-66	histone 3 [Camellia sinensis]	0.593648	0.243191	0.241255
gi|64213910|gb|AAY41234.1|	2.00E-55	putative peptidyl-prolyl cis-trans isomerase [Gossypium hirsutum]	0.296824	0.243191	0.482509
gi|71040673|gb|AAZ20285.1|	1.00E-67	ubiquitin fusion protein [Arachis hypogaea]	0.296824	0.972763	0.482509
gi|84620804|gb|ABC59516.1|	2.00E-57	chloroplast photosystem II 22 kDa component [Nicotiana benthamiana]	0.296824	0.729572	0.241255
gi|94502636|ref|YP_588293.1|	4.00E-41	chloroplast hypothetical protein [Zea mays subsp. mays]	5.342832	16.53696	0.482509

Frequency of a unigene = (Number of EST for a given unigene/the total number of EST of the library) ×1000.

Additional file 5: Expression of some selected genes in axillary buds of *D. nobile*.

Semi-quantization RT-PCR was used to monitor the changes of expression before and during the vernalization. Tubulin served as endogenous control to normalize the inputs of RNA templates. These results are in consistence with those from real-time qPCR to a large extent (Figure 4).

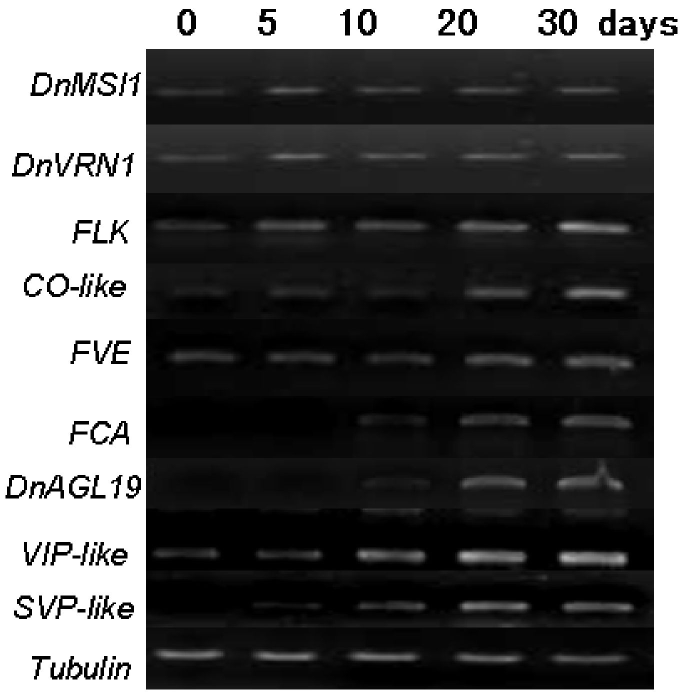

Primer pairs used in RT-PCR assay

Gene	Primer 1 (5'→3')	Primer 2 (5'→3')
MSI1	AACACGCCGTTCCTTTACG	GCCCTTCAATCTCAAATCAGG
VRN1	CGCTTATCATCTTCTCCACG	TGTTTCCGCTTTGTATCCTG
FLK	CATCCAGGAGTTTATTGCCG	GTAATCAGGTAGCCATCCGT
CO-LIKE	CGACATCCACTCCGCAAAT	GCCTGTTCTTCCTCTTCTCC
FVE	GGGCATAGAGATAAAGTGGTTG	ATTCGGGTTGCTGTGGAT
FCA	ACTCCACCGAATCAGCACCA	ACCAGTTCGCCAGTCCTTGA
AGL19	ACGGAGATGAGGCGGATAGA	GGCAGAGCAGGATTCAAGGT
VIP-LIKE	TGTTAGGGAGAGTGTAGGCA	ATTGGTGCGGAGAGAAGAC
SVP-LIKE	CCTATCCAAACAAGACCAACC	ATTTCCACGACCTGCTGTC

Additional file 6:

A

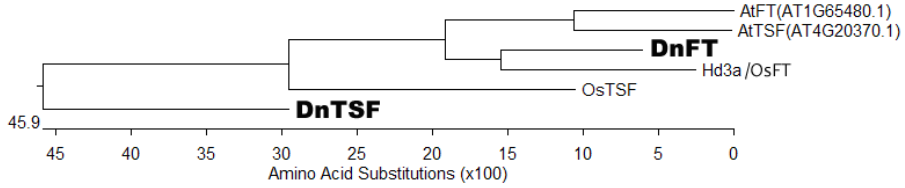

Percent Identity (columns) / Divergence (rows)

	1	2	3	4	5	6		
1		60.1	59.2	62.4	57.3	56.7	1	**DnTSF**
2	56.3		80.7	67.2	74.1	74.1	2	**DnFT**
3	58.2	22.4		64.9	71.4	71.4	3	Hd3a / **OsFT**
4	51.7	42.9	47.0		64.0	65.1	4	OsTSF
5	62.2	31.7	36.0	48.8		81.7	5	AtFT(AT1G65480.1)
6	63.5	31.7	36.0	46.7	21.0		6	AtTSF(AT4G20370.1)
	1	2	3	4	5	6		

B

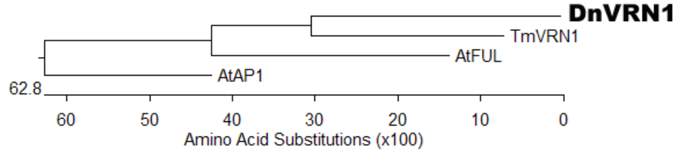

Percent Identity (columns) / Divergence (rows)

	1	2	3	4		
1		48.5	53.8	61.5	1	**DnVRN1**
2	84.0		54.2	51.9	2	AtAP1
3	70.2	69.3		56.1	3	AtFUL
4	53.7	74.9	64.9		4	TmVRN1
	1	2	3	4		

C

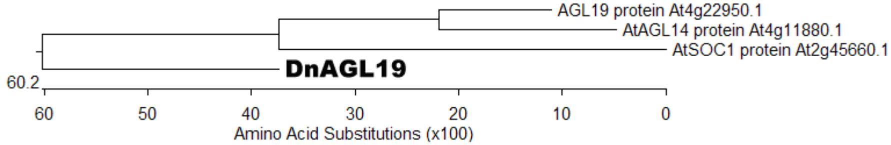

Percent Identity (columns) / Divergence (rows)

	1	2	3	4		
1		48.8	52.7	51.0	1	**DnAGL19**
2	83.1		57.1	53.5	2	AtSOC1 protein At2g45660.1
3	73.0	62.8		76.7	3	AGL19 protein At4g22950.1
4	77.3	70.9	27.9		4	AtAGL14 protein At4g11880.1
	1	2	3	4		

Additional file 7

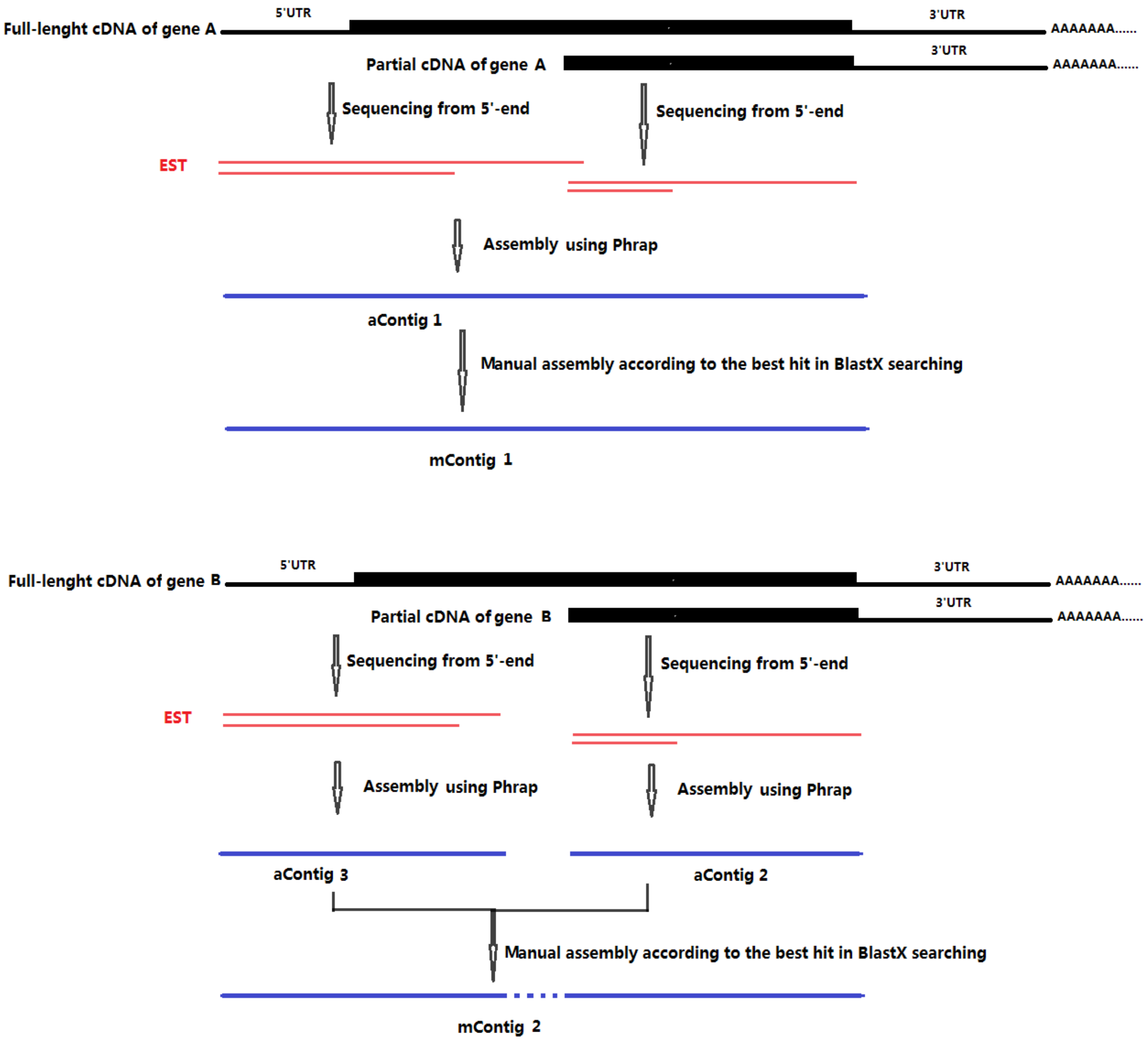

Supplement: Supplementary file 1 — (1) A scheme of sample pooling for library construction. (2) Primer pairs used in real-time qPCR assays for selected flowering-relevant genes. (3) Distributions of EST size of each library. (4) Venn diagram and a list of ESTs with possible roles in vernalization response. (5) Expression of selected genes in buds of D. nobile in response to vernalization treatment were assayed by semi-quantitative RT-PCR. Primer pairs were also listed. (6) Phylogenetic analysis for homologs of DnFT, DnVRN1 and DnAGL19 using MegAlign software embedded in the Lasergene package. Divergent distances are also shown. (7) A chart description of EST assembly. [file 757801.f1.pdf]
